# Cross‐sectional survey and surveillance for influenza viruses and MERS‐CoV among Egyptian pilgrims returning from Hajj during 2012‐2015

**DOI:** 10.1111/irv.12429

**Published:** 2016-11-11

**Authors:** Samir Refaey, Marwa Mohamed Amin, Katherine Roguski, Eduardo Azziz‐Baumgartner, Timothy M. Uyeki, Manal Labib, Amr Kandeel

**Affiliations:** ^1^ Egyptian Ministry of Health and Population (MOHP)CairoEgypt; ^2^ Influenza DivisionUS Centers for Disease Control and Prevention (CDC)AtlantaGAUSA

**Keywords:** Egypt, Hajj, influenza, MERS‐Coronavirus

## Abstract

**Background:**

Approximately 80 000 Egyptians participate in Hajj pilgrimage annually. The purpose of this study was to estimate influenza virus and MERS‐CoV prevalence among Egyptian pilgrims returning from Hajj.

**Study:**

A cross‐sectional survey among 3 364 returning Egyptian pilgrims from 2012 to 2015 was conducted. Nasopharyngeal (NP) and oropharyngeal (OP) swabs were collected from all participants. Sputum specimens were collected from participants with respiratory symptoms and productive cough at the time of their interview. Specimens were tested for influenza viruses, and a convenience sample of NP/OP specimens was tested for MERS‐CoV. Thirty percent of participants met the case definition for influenza‐like illness (ILI), 14% tested positive for influenza viruses, and none tested positive for MERS‐CoV. Self‐reported influenza vaccination was 20%.

**Conclusions:**

High prevalence of reported ILI during pilgrimage and confirmed influenza virus on return from pilgrimage suggest a continued need for influenza prevention strategies for Egyptian Hajj pilgrims. An evaluation of the Ministry of Health and Population's current risk communication campaigns to increase influenza vaccine use among pilgrims may help identify strategies to improve vaccine coverage.

## 1 Background

The Hajj pilgrimage to Mecca, Saudi Arabia, is among the largest pilgrimages in the world, with three million Muslims from over 180 countries participating every year, providing the potential for emerging epidemics.^1^ Acute respiratory tract infections are the most common diseases transmitted during Hajj, and influenza virus and rhinovirus are the most frequently detected respiratory viruses among pilgrims.^2^, ^3^

In addition, there is public health concern about the risk of human infections from Middle East respiratory syndrome coronavirus (MERS‐CoV) in Saudi Arabia. As of July 25, 2016, 1791 laboratory‐confirmed cases of human infection with MERS‐CoV from 26 countries were reported to the World Health Organization (WHO), including at least 640 deaths (CFR 36%), with the majority of cases reported in Saudi Arabia.^4^ Direct contact with dromedary camels has been implicated as a risk factor for zoonotic MERS‐CoV transmission, although the majority of MERS‐CoV outbreaks have been linked to exposures in healthcare settings.^5^, ^6^, ^7^ Although sustained MERS‐CoV transmission has not occurred to date, sporadic travel‐related clusters have been documented, leading to concern about potential MERS‐CoV dissemination during Hajj.^8^

Approximately 80 000 Egyptians participate in Hajj pilgrimage annually.^9^ In the wake of the 2009 H1N1 influenza pandemic, the Egyptian Ministry of Health and Population (MOHP) required pre‐departure vaccination against influenza A(H1N1)pmd09 for all pilgrims during the 2009 season.^9^ Since then, the MOHP has required seasonal influenza vaccination for all pilgrims as part of the Saudi visa application process. Although the requirement is not always enforced, seasonal influenza vaccine is available at local health offices for all Egyptian pilgrims throughout most of year. The MOHP has additionally conducted an annual survey among pilgrims returning from Hajj to explore the risk of influenza virus transmission to the broader community. Following the emergence of MERS‐CoV in Saudi Arabia, the MOHP expanded the survey to test for MERS‐CoV.

## 2 The Study

A cross‐sectional survey was conducted at Cairo International Airport among Egyptians returning from Hajj during the week following the end of Hajj each year from 2012 to 2015 (Table 1). Cairo airport was selected for this survey as it is the main point of entry into Egypt for returning Hajj pilgrims.^9^ Cairo airport receives 7‐8 flights during working hours (9am‐9 pm) from Saudi Arabia, accounting for approximately 1500‐2000 pilgrims per day.

**Table 1:** The distribution of Egyptian pilgrims surveyed by season according to gender, age group, presence of influenza‐like illness (ILI), vaccination status and influenza laboratory test result

Characteristics	2012N (%)N=824	2013N (%)N=740	2014N (%)N=827	2015N (%)N=973	OverallN (%)N=3364
Dates of Data Collection	3 Nov‐14 Nov 2012	22 Oct‐29 Oct 2013	11 Oct‐15 Oct 2014	29 Sept‐05 Oct 2015	
Male	401 (48.7)	360 (48.7)	426 (51.5)	429 (44.1)	1616 (48.0)
Age groups					
<5 y	0 (0)	1 (0.1)	2 (0.2)	6 (0.6)	9 (0.3)
5‐<15 y	0 (0)	0 (0)	0 (0)	0 (0)	0 (0)
15‐<50 y	215 (26.1)	255 (34.5)	222 (26.8)	217 (22.3)	909 (27.0)
50‐<65 y	446 (54.1)	369 (49.9)	475 (57.4)	577 (59.3)	1867 (55.5)
65+ y	163 (19.8)	115 (15.5)	128 (15.5)	173 (17.8)	579 (17.2)
Reported symptoms consistent with ILI	319 (38.7)	326 (44.1)	195 (23.6)	183 (18.8)	1023 (30.4)
Vaccinated	162 (19.7)	69 (9.3)	244 (29.5)	189 (19.4)	664 (19.7)
Laboratory‐confirmed influenza	145 (17.6)	105 (14.2)^a^	133 (16.1)	101 (10.4)^b^	484 (14.4)
Influenza A (H1N1)	42 (5.1)	22 (3.0)	3 (0.4)	50 (5.1)	117 (3.5)
Influenza A (H3N2)	54 (6.6)	38 (5.1)	60 (7.3)	35 (3.6)	187 (5.6)
Influenza B	49 (6.0)	44 (6.0)	70 (8.5)	17 (1.8)	180 (5.4)
Tested for MERS‐CoV^c^					
Sputum	25 (3.0)	60 (8.1)	100 (12.1)	79 (8.1)	264 (7.8)
NP/OP	187 (22.7)	740 (100)	823 (99.5)	187 (19.2)	1937 (57.6)

a One influenza A‐positive specimen was not subtyped.

b One specimen tested positive for both A(H1N1) and A(H3N2).

c All MERS‐CoV tests were negative. All sputum specimens were paired with NP/OP specimens.

A team from the MOHP sought to enroll a convenience sample of approximately 10% of pilgrims from each flight returning from Hajj and congregating at the airport carousels, regardless of age, sex, and illness status. After providing verbal consent, participants were asked about demographic information, respiratory symptoms, and whether they received vaccines as part of their Hajj visa application process. Both nasopharyngeal (NP) and oropharyngeal (OP) swabs were collected from all participants regardless of the presence of respiratory symptoms. Sputum specimens were collected from participants who presented with respiratory symptoms and a productive cough at the time of their interview. Travelers who reported a history of subjective fever (a proxy for measured fever) and cough with symptom onset in the previous 10 days were categorized as having influenza‐like illness (ILI).^10^ For minors, consent and survey responses were provided by accompanying parents.

Samples were stored in viral transport media (VTM) on liquid nitrogen and transferred to the MOHP's Central Public Health Laboratory on the day of collection. All NP/OP specimens were tested by real‐time RT‐PCR (rRT‐PCR) for the presence of influenza A and B viruses. Specimens positive for influenza A were subsequently tested for influenza A virus subtypes. Sputum specimens and a convenience sample of NP/OP specimens were tested for MERS‐CoV according to WHO guidelines.^11^ The proportion of samples testing positive for influenza virus from participants was compared to those collected from ILI case‐patients through the national surveillance system during the same time period.

**Figure 1:**
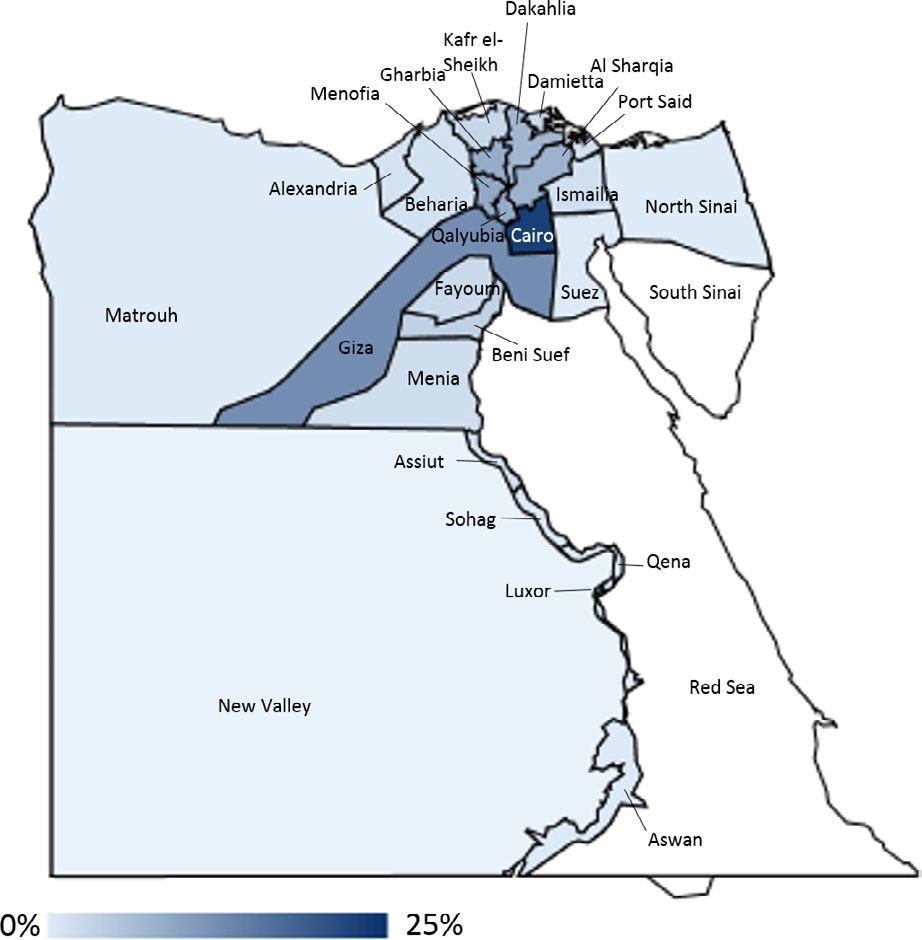
The geographical distribution for the governorate of origin of Egyptian pilgrims surveyed from 2012 to 2015

During the 2012‐2015 seasons, 3559 pilgrims were approached for participation; 3364 (94.5%) provided consent for participation and swab collection. An average of 135 pilgrims was interviewed daily for 5‐8 days each year. The median age among participants was 56 years (range 0‐105), and 56% of participants were aged 50‐64 years (Table 1). Approximately half (1616 [48%]) of the participants were male. Surveyed pilgrims resided in 25 of the 27 governorates of Egypt, although the majority were from Cairo, Giza, Menofia, and Qalyubia (774 [23%], 423 [12.6%], 305 [9.1%], and 292 [8.7%] respectively) (Figure 1).

Self‐reported influenza vaccination among participants was low across seasons, with an overall vaccination coverage of 19.7% throughout the study period. There was no difference in influenza vaccination coverage by age (median of 56 years among unvaccinated participants and 55 years among vaccinated participants, *P*=0.4) or by sex (19.5% females and 20.0% males, *P*=0.7).

Thirty percent of participants (1023/3364) reported symptoms consistent with ILI during their travel. Approximately a fifth of those with ILI symptoms tested positive for influenza virus (217/1023; 21.2%). An additional 20.7% of participants with at least one reported respiratory symptom (242/1170) and 11.6% of participants without any of the four symptoms captured in the questionnaire (i.e., feverishness, cough, sore throat, and dyspnea) (141/1216) tested positive for influenza virus. There was no difference in the proportion of persons who were influenza virus positive by sex (14.7% females and 14.1% males, *P*‐value=0.6) or by age (median of 55 years among influenza virus‐positive participants and 56 years among influenza virus negative participants, *P*=0.9). The proportions of influenza virus‐positive specimens among participants meeting the ILI case definition and among those collected through the national influenza surveillance system (hospitalized and outpatient ILI) were similar in 2012 and 2013 (2012: 26.0% vs 20.2% (*P*=0.1); 2013: 17.5% vs 17.3% (*P*=1.0), respectively), higher among participants in 2014 (28.2% among participants, 16.1% among those tested through the influenza surveillance system, *P*=0.008), and lower among participants in 2015 (12.0% among participants, 24.2% among those tested through the influenza surveillance system, *P*=0.006). MERS‐CoV testing was conducted on NP/OP specimens for 1673 of 3364 participants (49.7%) and on paired sputum and NP/OP specimens for 264 (7.8%) of participants; none tested positive for MERS‐CoV.

## 3 Conclusions

Thirty percent of surveyed Hajj pilgrims reported symptoms consistent with ILI at some point during their pilgrimage and over 14% tested positive for seasonal influenza viruses on their return. The high prevalence of ILI during pilgrimage and confirmed influenza virus on the pilgrims' return to Egypt suggests a continued need for influenza prevention strategies for Egyptian Hajj pilgrims. These findings are higher than those of prior surveys where influenza virus positivity was 1.3%‐7.8% among returning Hajj pilgrims regardless of clinical status.^12^, ^13^ Differences between our results and those of other studies might be due to subtle differences in sociodemographic characteristics including age distribution and prior vaccination among those surveyed. Findings from this study may additionally be biased by the non‐random selection of participants and recall bias.

While MERS‐CoV was not detected in any respiratory swabs or sputum specimens collected from participants, no information regarding health care or camel exposure was collected, limiting the interpretation of these results. In future iterations, the MOHP could consider adding exposure questions to better understand the risk of MERS‐CoV infection among Egyptians on Hajj. Additionally, upper respiratory tract specimens have been shown to yield significantly lower MERS‐CoV genome loads than lower respiratory tract specimens (such as tracheal aspirates), which may have limited our ability to detect MERS‐CoV.^14^

Despite the MOHP's requirement of influenza vaccination for all Egyptian Hajj pilgrims, only 19.7% of participants reported receiving seasonal influenza vaccine prior to their pilgrimage. This figure may underestimate true influenza vaccine coverage, as interviewers only asked about vaccines received as part of Hajj travel preparations. Nevertheless, further health education through local media may help raise awareness about the value of influenza vaccination prior to departure for Hajj, especially among older age groups at high risk of influenza complications.^15^ An evaluation of the MOHP's current risk communication campaigns to increase influenza vaccination use among pilgrims may help identify strategies to improve influenza vaccine coverage.

## Conflict of Interest

Co‐authors have no conflicts of interest to declare.

## Disclaimer

The findings and conclusions in this report are those of the author(s) and do not necessarily represent the official position of the Centers for Disease Control and Prevention.
